# Characterization of two new monoclonal antibodies against human papillomavirus type 16 L1 protein

**DOI:** 10.1186/1746-1596-9-101

**Published:** 2014-05-29

**Authors:** Yan Wang, Qinglong Shang, Weizhen Xu, Di Li, Hongxi Gu, Lanlan Wei

**Affiliations:** 1 Department of Microbiology, Heilongjiang Key Laboratory of Infection and Immunity, Harbin Medical University, Harbin 150081, China; 2 Pathogenic-Biological Key Laboratory of Heilongjiang Province, Harbin Medical University, Harbin 150081, China

**Keywords:** Human papillomavirus, Monoclonal antibody, Variable regions

## Abstract

**Background:**

Human papillomavirus type 16 (HPV16) infection is implicated in cervical carcinogenesis. This study aimed to characterize two new monoclonal antibodies (mAbs) against HPV L1 protein.

**Methods:**

The immunocompetence of AE3 and AG7 mAbs for HPV L1 protein was evaluated by Western blot analysis, immunostaining, hemagglutination inhibition assay, and ELISA. The heavy chain variable region (VH) and light chain variable region (VL) of AE3 and AG7 mAbs were sequenced and analyzed.

**Results:**

Both mAbs specifically recognized HPV16 L1 and virus-like particles (VLPs). Both the affinity and the titer of AE3 mAb were higher than that of AG7. There were differences in sequences in the complementary determining regions (CDR) 2 and 3 of VL, as well as in the CDR1 and CDR3 of VH. The two mAbs have distinct predicted three-dimensional structures.

**Conclusions:**

We characterized two mAbs neutralizing antibodies for HPV L1 protein, which would help develop genetic-engineered neutralizing antibodies against HPV16 for diagnostic and therapeutic purposes.

## Background

Human papillomaviruses (HPVs) are non-enveloped, epitheliotropic viruses that mainly cause abnormal hyperplasia in the skin and mucosa. HPVs are related to a variety of benign and malignant lesions [1]. HPV type 16 (HPV16) has been found as a high risk of carcinogenesis [2-4].

Numerous studies have been preformed to develop HPV vaccine [5]. However, current HPV vaccine fails to completely prevent against cervical cancer as the cervical cancer is related to more than 15 types of HPV infection [6]. Early diagnosis and treatment of high risk HPV-infection that may induce pathological changes are still keys to control cervical cancer and precancerous lesions [7,8]. Neutralizing antibodies against HPV L1 protein have shown promise in the prevention of cervical cancer [9]. The L1 gene of HPV is well-conserved and encodes a major structural protein of HPV16. The L1 protein contains a number of epitopes that can induce the production of specific neutralizing antibodies against HPV [10,11]. Therefore, the generation and selection of highly efficient anti-HPV L1 monoclonal antibodies (mAbs) are crucial for the clinical diagnosis and treatment of HPV infection.

In this study, the recombinant HPV16 L1 protein was used as an antigen to generate two mAbs. To analyze the differences in these two mAbs, the sequences of heavy chain variable region (VH) and light chain variable region (VL) were determined and compared between two mAbs. Our results provide important information on the development of HPV neutralizing antibodies.

## Methods

### Preparation of mAbs

Generation of recombinant HPV16 L1 protein was induced by isopropy-β-D-thiogalactoside (IPTG) from pQE31-HPV16L1/M15 constructed in our laboratory. Purified protein was used to immunize BALB/c mice, and two hybridoma cell lines (AE3 and AG7) were selected which stably expressed neutralizing antibodies against HPV16 L1 protein. The culture supernatant and ascites from mice carrying AE3 and AG7 hybridomas were purified by caprylic acid-ammonium sulfate methods.

### Western blot analysis

HPV16 E6 and HPV16 E7 proteins were produced and purified using a baculovirus expression system. Fifty microgram aliquots of total protein were separated on 12% SDS-polyacrylamide gels and transferred onto nitrocellulose membranes. The membranes were blocked with TBST buffer containing 5% skim milk and incubated with AE3 or AG7 mAb (1:1000) overnight. After washing, the membranes were incubated with horseradish peroxidase-conjugated anti-mouse IgG (Santa Cruz Biotechnology, Santa Cruz, CA), and the protein bands were visualized by enhanced chemiluminescence (ECL).

### Immunofluorescence

Sf9 cells were infected with HPV recombinant baculovirus rBacV/HPV16 L1 on coverslips in 6-well plates. Three days later, the cells were fixed in 10% acetic acid, 50% ethanol, washed with phosphate-buffered saline (PBS) and then incubated with the mAbs of AE3 and AG7 for 1 h at 37°C followed by incubation with FITC-conjugated secondary antibody (1:80) (Invitrogen, USA). The fluorescence was detected under an Olympus AX70 epifluorescence microscope (Olympus, Tokyo, Japan).

### Immunoelectron microscopy

HPV16 L1 VLP crude extract was incubated with purified mAb at 37°C for 1 h and then at 4°C overnight. The mixture was centrifuged at 12,000 g for 90 min, and then the supernatant was removed. In the negative control, 3% phosphotungstic acid and 400-mesh high-transmission nickel grids were used. VLPs were observed under a Hitachi H600 transmission electron microscope.

### Hemagglutination inhibition (HI) assay

Red blood cells (RBCs) from BALB/c mice were diluted in 0.1% BSA-PBS to 1%. Ascites were mixed with the RBC precipitation of equal volume followed by incubation at 4°C overnight. The supernatant was collected after centrifugation at 1,000 g for 5 min and then incubated at 56˚C to inactivate the complements. The supernatant was mixed with HPV16 VLPs of equal volume and loaded into a 24-well plate followed by incubation at 37°C for 1 h. Next, 1% RBC of equal volume was added and the mixture was further incubated for 3 h at room temperature before evaluating the HI titer.

### ELISA

The titers of mAbs (AE3 and AG7) in the supernatants of cultured hybridoma cells and ascites were analyzed by indirect ELISA. Briefly, ELISA plates were coated with HPV16L1 protein at 4°C overnight. After washing with PBS and blocking with 1% BSA, the plates were incubated with serially diluted mAbs followed by incubation with horseradish peroxidase conjugated anti-mouse IgG for 1 h. After washing with PBS, the 3,3′,5,5′-Tetramethylbenzidine substrate (100 μl/well; Santa Cruz) was added followed by incubation for 15 min. The reaction was stopped by the addition of 1 M HCl (100 μl/well), and the absorbance was determined at 490 nm.

### Sequencing of VH and VL genes

Two hybridoma cell lines (AE3 and AG7) in logarithmic growth phase were harvested, and total RNA was extracted with TRIzol (Invitrogen, Carlsbad, CA) according to the manufacturer’s instructions. cDNA was synthesized with total RNA and labeled with polyG at the 3′ end of the RNA using TdT enzyme (Takara, Dalian, China). RT-PCR was performed using a RT-PCR kit (Takara) and primers were as follows: for VL gene P1 (5′-CCCCCCCCCCCCCCC-3′) and P2 (5′-GCGCCGTCTAGAATTAACACTCATTCCTGTTGAA-3′); for VH gene P1 (5′-CCCCCCCCCCCCCCC-3′) and H2 (5′-ACCTTAGGAGTCAGAGTAATGGTGAGCACATCC-3′). PCR conditions were denaturation at 95°C for 4 min, 35 cycles of denaturation at 94°C for 40 s, annealing at 55°C for 45 s and extension at 72°C for 1 min, and a final extension at 72°C for 7 min. PCR products were purified for sequencing. The nucleotide and amino acid sequences of AE3 and AG7 were compared and analyzed using the IgBLAST software ( http://www.ncbi.nlm.nih.gov). Three dimensional (3D) structure prediction was performed using Geno3D ( http://geno3d-pbil.ibcp.fr).

## Results

### Immunocompetence of AE3 and AG7 mAbs

**Figure 1:**
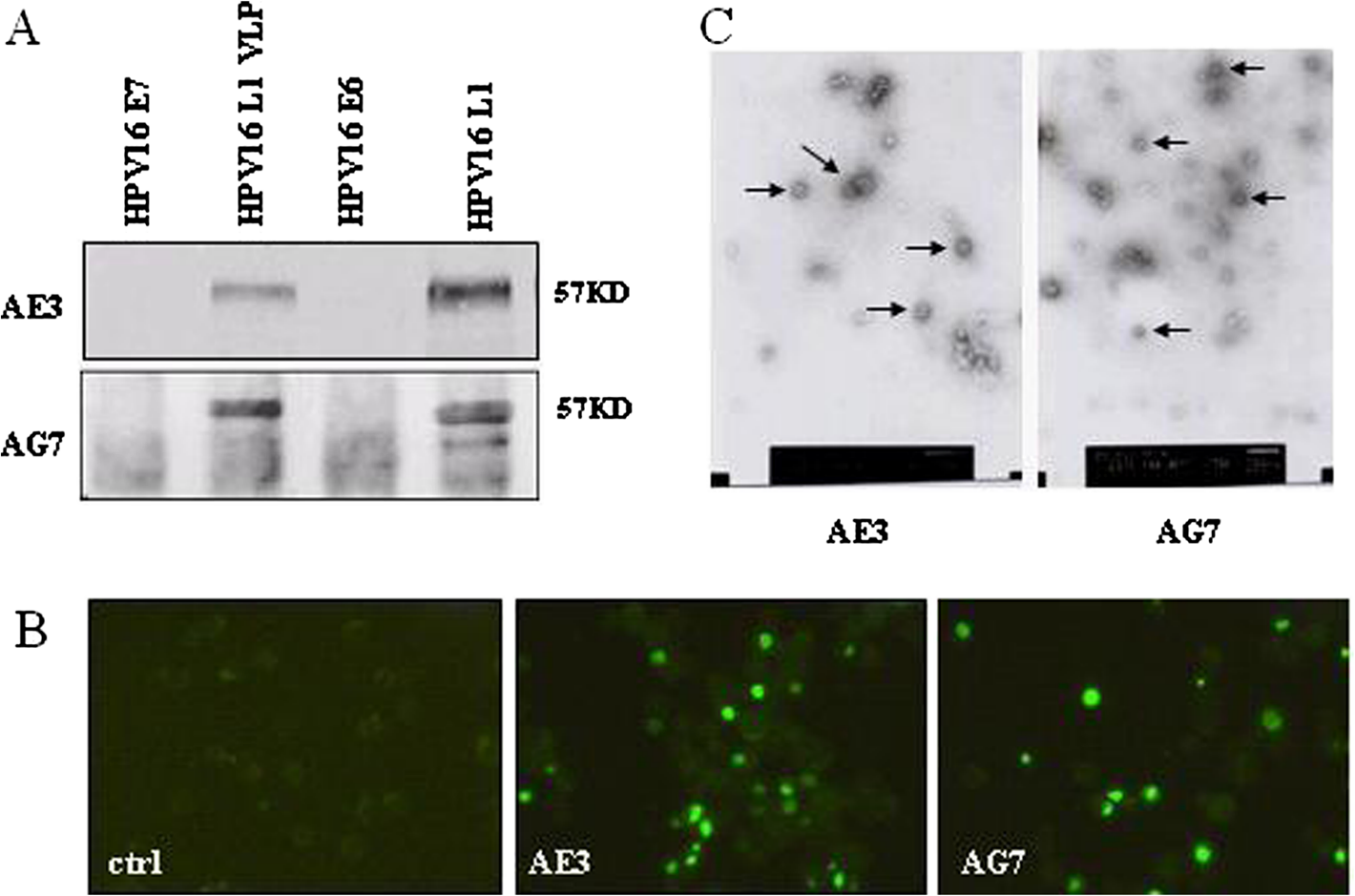
**Immunocompetence of AE3 and AG7 mAbs. A**: Western blot analysis of the specificity of AE3 and AG7 mAbs (1:1000) to different antigens. **B**: Sf9 cells were inoculated with rBacV/HPV16 L1 in 6-well plates. The supernatant of cultured AE3 and AG7 was incubated for 1 h at 37°C followed by incubation with FITC-conjugated secondary antibody (1:80). Immunofluorescence was detected under an epifluorescence microscope (×200). **C**: The images of HPV16L1 VLPs under an immunoelectron microscope. The morphology was indicated by the arrows (×5,000).

Western blot analysis showed that AE3 and AG7 mAbs could specially bind to HPV16 L1 VLP and HPV16 L1 proteins, but not to HPV16 E6 and HPV16 E7 proteins, indicating the specificity of two mAbs (Figure 1A). Immunostaining revealed that AE3 and AE7 could recognize the virus-infected Sf9 cells (Figure 1B). HPV16 L1 VLPs were a spherical particle with 55-nm in diameter (Figure 1C). The HI titer of AE3 and AG7 mAbs was 1:64 and 1:8, respectively. The titers of AE3 and AG7 mAb was 1:5120 and 1:80, respectively, in culture supernatant and 1:10^6^ and 1:10^4^, respectively, in ascites (Table 1).

**Table 1:** Comparison of AE3 and AG7 mAbs

	**AE3**	**AG7**
Titer of ascites	1:10^6^	1:10^4^
Titer of supernatant	1:5120	1:80
Concentration of mAb	148 μg/mL	480 μg/mL
Relative affinity	0.075 μg/mL	0.75 μg/mL
HI titer	1:64	1:8

### Detection of VH and VL genes of AE3 and AG7 mAbs

**Figure 2:**
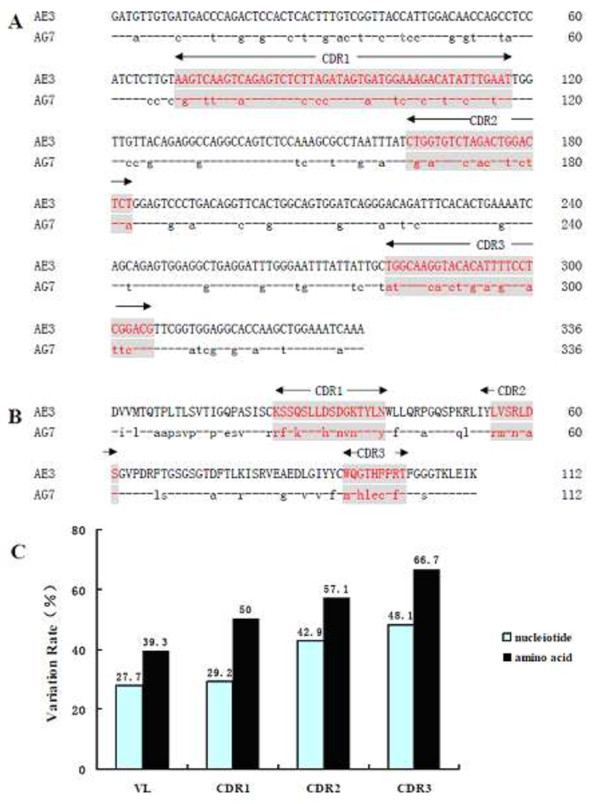
**Comparison of nucleotides and amino acids of VL between AE3 and AG7 mAbs. A**: Comparison of nucleotides of VL between AE3 and AG7 mAbs. **B**: Comparison of amino acid of VL between AE3 and AG7 mAbs. **C**: Variation rate of VL in AE3 and AG7 mAbs. The variation rate of two selected sequences indicated the ratio of different nucleotide or amino acid to total nucleotide or amino acid.

**Figure 3:**
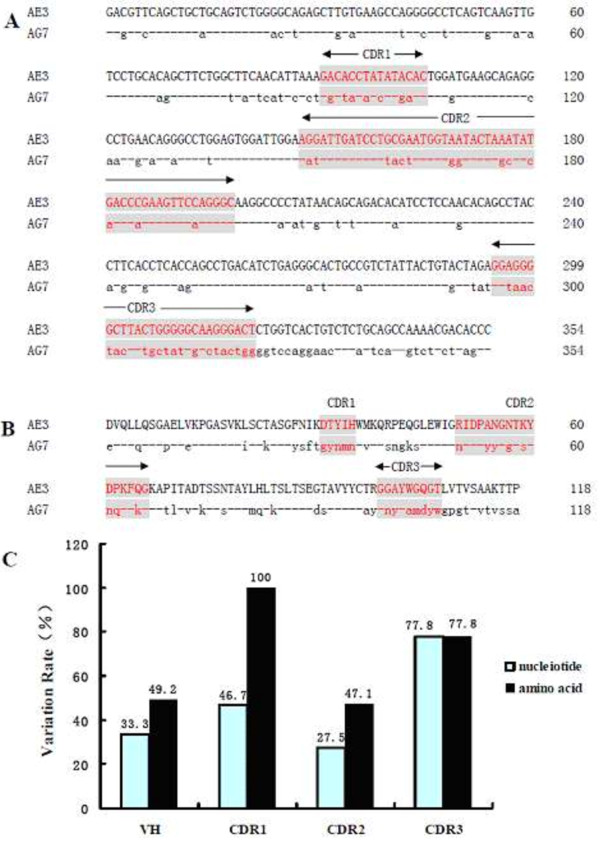
**Comparison of nucleotides and amino acids of VH between AE3 and AG7 mAbs. A**: Comparison of nucleotides of VH between AE3 and AG7 mAbs. **B**: Comparison of amino acids of VH between AE3 and AG7 mAbs. **C**: Variation rate of VH of AE3 and AG7 mAbs.

**Figure 4:**
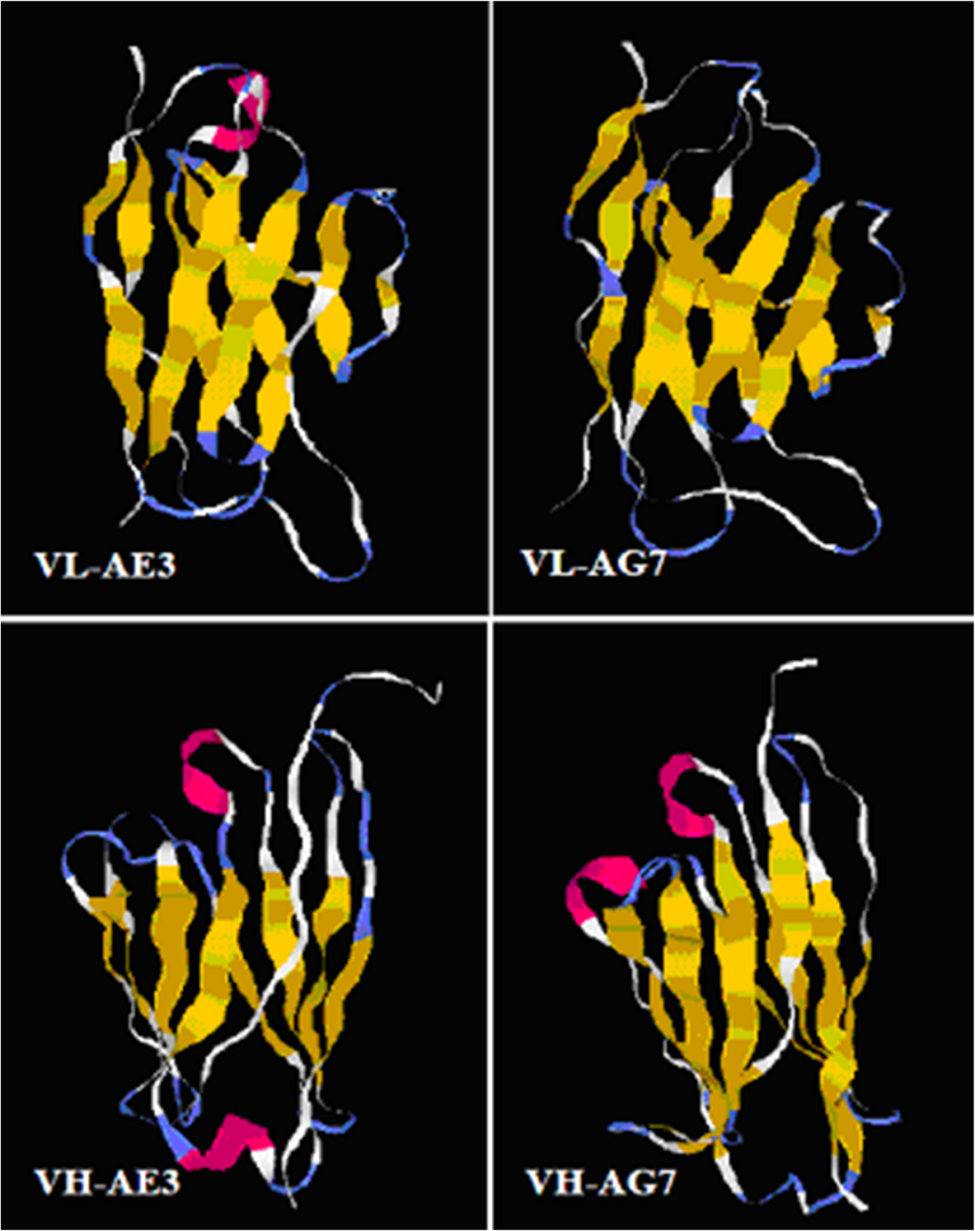
**Three-dimensional structure prediction of VL and VH of AE3 and AG7.** The light chain of AE3 mAb had one α-helix (red), but the light chain of AG7 had no α-helix. There were two α-helies (red) at both sides of the heavy chain of AE3 mAb, while the two α-helies of heavy chain of AG7 (red) were located at the same side.

The VL genes of AE3 and AG7 mAbs each included 336 nucleotides (nts) (Figure 2A), and encoded 112 amino acids (aas) (Figure 2B). The VL had 4 framework regions (FRs) and 3 complementary-determining regions (CDRs), and the VL-specific cysteine residues (aa23 and aa93) located in one of the FRs, in accordance with the characterization of mouse VL gene. The comparison of VL between two mAbs was shown in Figure 2C. The variation rate of VL in two mAbs was 27.7% at the nt level, including 29.2% (14/48) in the CDR1, 42.9% (9/21) in the CDR2, and 48.1% (13/27) in the CDR3. The variation rate of VL in two mAbs was 39.5% at the aa level, including 50% (8/16) in the CDR1, 57.1% (4/7) in the CDR2, and 66.7% (6/9) in the CDR3. In addition, the CDR2 and CDR3 regions had higher variation rate (Figure 2C).The VH genes of AE3 and AG7 mAbs each contained 354 nucleotides (Figure 3A), and encoded 118 amino acids (Figure3B). Similar to VL, each VH had 4 FRs and 3 CDRs. The VH protein contained 2 characteristic cysteine residues (aa22 and aa96), consistent with the characteristics of mouse VH gene. The difference in homology between two mAbs was shown in Figure 3C. The variation rate of VH regions was 33.3% (118/354) at the nt level, including 46.7% (7/15) in the CDR1, 27.5% (14/51) in the CDR2, and 77.8% (21/27) in the CDR3. At the aa level, the variation rate was 49.2% (58/118), including 100% (5/5) in the CDR1, 47.1% (8/17) in the CDR2, and 77.8% (7/9) in the CDR3. The highest variation rate at nt and aa levels was in the CDR3 for VH genes, but CDR1 also showed an increased variation rate between two mAbs (Figure 3C).3D structure predictions of VL of each mAb showed an alpha helix in the secondary structure of AE3, which was absent in AG7. For VH, two alpha helices were predicted in the secondary structure of AE3 located at two different sides of the protein, but two alpha helices were predicted in the secondary structure of AG7 located at at the same side of the protein (Figure 4).

## Discussion and conclusions

In the present study, we constructed and compared two mABs for HPV16 L1. The immune activity of AE3 mAb was stronger than that of AG7. In addition, the correlation between the differences in the biological activity of two mAbs and their gene sequences was evaluated. CDR3 encoded by the VH gene of two mAb had 9 amino acids, which suggested that the corresponding antigens were relatively complex [12]. The VH of AE3 mAb was rich in serine, threonine, and asparagine residues, which are potential glycosylation sites [13]. Although the VH of AG7 mAb had a greater number of variant amino acids compared to the VH of AE3 mAb, there were still some serine, threonine, and asparagine residues in the VH of AG7 mAb, which may enhance the hydrophilicity and the affinity of the antibody to corresponding antigen [14].

CDR is an antigen-specific binding site, and anti-idiotypic antibodies represent their antigenicity. CDR2 and CDR3 not only affect the affinity of antibodies, but have the functions of FR-1 to assist antibody folding [12]. Especially, the heavy-chain CDR3 is the site for the specific binding between antibodies and antigens [15]. In this study, the function of VLs and VHs of two mAbs was similar, but the amino acids of these 2 regions varied, especially in the CDRs 1–3. The variation in the nucleotide and amino acid sequences of VL and VH of two mAbs was rather high in CDR2 and substantially higher in CDR3. The variation in CDR3, which affects the affinity (heavy-chain: 77.8%; light-chain: 66.7%), is postulated to attribute to the main difference between the characteristics of AE3 and AG7. Therefore, the different antigenic determinants of these two mAbs indicate that the mAbs target different epitopes.

Comparisons of the spatial structures of similar proteins showed that the 3D structure of a protein was more conservative than the primary structure, but the latter was more conservative than DNA sequence. Generally, the 3D structure of a protein determines its function, and the structure of VH and VL of a specific antibody determines its affinity and specificity. However, the function of some antibodies largely depends on the VH, but on the VL to a lesser extent. No studies have reported the simulated 3D structure of anti-HPV16 L1 antibodies. In this study, the homology modeling methods were employed to simulate the 3D structures of VL and VH of both AE3 and AE7 mAbs. Our results showed that VL structure of AE3 had more α-helix in the secondary structure compared to that of AG7; in the VH structure, two α-helies were located at opposite sides of AG3, while they were located at the same side of AG7. We hypothesized that the differences in their structures are related to the different affinity to antigens, but further studies are required to elucidate the importance of these differences.

For future studies, bioinformatics techniques can be used to establish the 3D structure of the single-chain variable fragment (scFv) of these mAbs. A scFv model can be optimized with respect to the mechanical motions and kinetic parameters to potentially identify the functional role of each variable region in these mAbs. These studies will provide a theoretical basis for rationally designing high-affinity antibodies for the application in the diagnosis and treatment of HPV infection.

## Competing interests

The authors declare that they have no competing interests.

## Authors’ contributions

YW and QS designed and performed the experiments, WX and DL participated in the experiments, HG and LW supervised the study and drafted the manuscript. All authors read and approved the final manuscript.
